# Integrating causal discovery and clinically-relevant insights to explore directional relationships between autistic features, sex at birth, and cognitive abilities – ERRATUM

**DOI:** 10.1017/S0033291725001114

**Published:** 2025-05-29

**Authors:** Angela Tseng, Sunday M. Francis, Eric Rawls, Christine Conelea, Nicola M. Grissom, Erich Kummerfeld, Sisi Ma, Suma Jacob

**Affiliations:** 1 Division of Child&Adolescent Psychiatry, Semel Institute of Neuroscience and Human Behavior, University of California Los Angeles, Los Angeles, CA, USA; 2 Department of Psychiatry and Behavioral Sciences, University of Minnesota, Minneapolis, MN, USA; 3 Department of Psychology, University of Minnesota, Minneapolis, MN, USA; 4 Institute for Health Informatics, University of Minnesota, Minneapolis, MN, USA

When this article was originally published in Psychological Medicine it omitted to highlight the equal authorship of Angela Tseng and Sunday M. Francis. This article has been updated so that this is included.

The publisher apologises for this error.
